# *Lactiplantibacillus plantarum* Postbiotics: Alternative of Antibiotic Growth Promoter to Ameliorate Gut Health in Broiler Chickens

**DOI:** 10.3389/fvets.2022.883324

**Published:** 2022-07-04

**Authors:** Hui Mei Chang, Teck Chwen Loh, Hooi Ling Foo, Eric Teik Chung Lim

**Affiliations:** ^1^Department of Animal Science, Faculty of Agriculture, Universiti Putra Malaysia, Serdang, Malaysia; ^2^Institute of Tropical Agriculture and Food Security, Universiti Putra Malaysia, Serdang, Malaysia; ^3^Department of Bioprocess Technology, Faculty of Biotechnology and Biomolecular Sciences, Universiti Putra Malaysia, Serdang, Malaysia; ^4^Institute of Bioscience, Universiti Putra Malaysia, Serdang, Malaysia

**Keywords:** *Lactiplantibacillus plantarum*, postbiotics, antibiotic growth promoter, gut health, mucin dynamics, immune response, broiler chickens

## Abstract

The postbiotic produced from *Lactiplantibacillus plantarum* has been revealed as a potential alternative to antibiotic growth promoters (AGP). It helps to stimulate growth performance, improve nutrient digestibility, intestinal histomorphology, immune response, and improve meat quality in livestock. However, there is a paucity of information on the effects of *L. plantarum* postbiotic produced by formulated media on the gut health and immune response. Therefore, this study was conducted by using three strains of dietary *L. plantarum* postbiotics to determine the growth performance, intestinal histomorphology, intestinal mucin production, and immune status in broiler chickens. A 245 male Cobb 500-day-old birds were assigned randomly to five treatments, namely, NC: basal diet only (negative control), OTC: basal diet + 0.01% (w/w) oxytetracycline (positive control), RG11: basal diet + 0.1% (v/w) Postbiotic RG11, RI11: basal diet + 0.1% (v/w) Postbiotic RI11, and RS5: basal diet + 0.1% (v/w) Postbiotic RS5. The body weight and feed intake were taken weekly. The small intestine and its mucus, ceca digesta were collected on days 21 and 42. Fresh excreta for crude mucin production were collected 3 days before slaughter on day 42. From the findings, RS5 recorded a significant highest (*p* < 0.05) final body weight, body weight gain, and significant lowest (*p* < *0.05*) feed conversion ratio. The concentrations of glutathione peroxidase, superoxide dismutase (SOD), acidic mucin, sulfated mucin, and intestinal trefoil factor were significantly higher (*p* < 0.05) in the birds fed with RI11 and RS5. Postbiotics RI11 and RS5 had up-regulated expression of intestinal Mucin 2, occludin, and secretory immunoglobulin A. The antibiotic-fed chickens also showed a reduced (*p* < 0.05) total bacteria and *Bifidobacterium* population but a significantly increased (*p* < 0.05) the population of *Escherichia coli* in the jejunum. In conclusion, the supplementation of *L. plantarum* postbiotic can be used to substitute AGP as it promoted growth performance, mucin production, ameliorated tight junction permeability, and immune status in broiler chickens due to improved gut health and beneficial bacteria colonization.

## Introduction

Production (performance), wellbeing, and welfare of the livestock are the ultimate concerns for a profitable business in the poultry industry. The increment of inflammation incidence, heat stress, dysbiosis, and genetic selection had contributed to low-grade inflammation, which eventually leads to diseases and pathogenic infections. One of the effective strategies to mitigate these adverse effects is to ameliorate gastrointestinal health, especially through immunomodulation of gut microbiota, mucin dynamics, and reinforcement of the intestinal barrier. Since birth, the gut microbiota starts to protect the bird by producing and releasing compounds such as short-chain fatty acids (SCFAs), bacteriocin, and lipopolysaccharides into the intestine against pathogens (1) despite being subjected to changes with time (2). As the gut becomes more matured, the mucins such as acidic and sulfated mucins, together with tight junction proteins, work synergistically with immune-related compounds such as pro- and anti-inflammatory cytokines and immunoglobulin A to establish a robust gut (2). Meanwhile the intestinal trefoil factor is the key indicator of intestinal maturation and inflammation (3). On the other hand, the gut is an intricate and dynamic area due to continuous exposure to the external environment and various potential stressors. Therefore, farmers used various feed additives or supplements to boost animal performance and health.

Antibiotic growth promoter (AGP) has been used to enhance feed conversion ratio, and growth performance (3–6), and reduce bacterial infection (7–9) despite the actual underlying mechanism that has remained unclear. Accumulating works of literature proved that the prolonged usage of AGP resulted in the emergence of antibiotic resistance genes in the ecosystem, particularly through antimicrobial residue in the food chain (10, 11), and reduced its efficacy (12). The limitation and ban of in-feed inclusion of antibiotic growth promoters have sped up the momentum to look for potential and safe alternatives to surmount its adverse effects. Hence, it is necessary to use another alternative for disease prevention and stimulate the growth performance in commercial broiler production.

The *Lactiplantibacillus plantarum* (formerly, known as *Lactobacillus plantarum*) (13) was isolated from fermented Malaysian food (14, 15). A recent *in vitro* study reported that *L. plantarum* postbiotics used in this study contain various beneficial organic acids, including SCFA, pyrrole compounds, intermediary compounds, and bacteriocin, which exhibit antimicrobial and antioxidant activities (16). These beneficial functional properties, in turn, lead to anti-inflammatory activity once supplemented in feed. This was proven by the studies conducted on various livestock to improve growth performance, meat quality, and antioxidant activities of blood plasma, gut permeability, and immune response. Moreover, the supplementation of dietary postbiotics also reduced the *Escherichia coli, Enterobacteriaceae*, and *Salmonella* population in the caecum and mitigated the effects due to heat stress in broiler chickens (17). Furthermore, the feed additive should improve nutrient availability, reduce pathogenic microbial growth to produce safe food for human consumption, and minimize negative environmental implications for sustainable farming (18).

Extensive studies have been conducted on the implications of the supplementation of postbiotics on the livestock, yet little information is available on the gut microbiota immunomodulation, mucin dynamics, and immune response. The recent research focus has shifted to the importance of gut health, and gut microbiota on the overall health status of the animal as the gut is responsible for nutrient digestion and assimilation, protective barrier against exterior pathogens by secreting immune-related compounds besides acting as a reservoir for diverse microbes (19). In the light of the information presented above, we evaluated the potential of postbiotics originating from *L. plantarum* to modulate gut microbiota, mucin dynamics, and immune response in broiler chickens compared with antibiotic growth promoters. To complete the objective mentioned, several parameters were studied such as the antioxidant concentration (superoxidase dismutase, glutathione peroxidase, and malondialchehyche), intestinal trefoil factor and type of mucin as those are the key factors to determine the gut health of the birds.

## Materials and Methods

### Bacterial Maintenance and Postbiotic Production

The *L. plantarum* RG11, RI11, and RS5, which were previously isolated from the local traditional Malaysian fermented food, were obtained from the Laboratory of Biotechnology, Department of Bioprocess Technology, Faculty of Biotechnology and Biomolecular Sciences, Universiti Putra Malaysia. The stock cultures kept at −20°C were revived twice using de-Mann Rogosa Sharpe (MRS) broth and incubated at 30°C for 48 and 24 h as described by Foo et al. (14). Then, the active bacteria were washed once with sterile 0.85% (w/v) NaCl (Merck, Darmstadt, Germany) solution and adjusted to 10^9^ CFU/ml before being inoculated into formulated media and incubated at 30°C for 24 h according to the method described by Mohamad Zabidi et al. (20). Finally, the postbiotic produced was ready to be used after centrifuging at 10,000 *g* for 15 min at 4°C and filtered through 0.22-μm cellulose acetate membrane (Sartorius Minisart, Germany) to remove all the viable bacterial cells. The *L.plantarum* strains selected for this study were based on the results obtained from previous research conducted by Chang et al. (16). The postbiotics RG11, RI11, and RS5 contain 15.4–17.5 mM acetic acid, 15.1–23.8 mM caproic acid, 30.8–426 g/L lactic acid, 31.9–36.2% hydroxyl radical scavenging activity, and 1.90–2.25 mg/L of ascorbic acid-reducing activity and exhibited inhibitory activity against positive indicator bacteria and pathogenic bacteria.

### Experimental Design, Animals and Housing Management

A total of 245 male Cobb 500-day-old chickens were bought from a local hatchery. On arrival, all the birds were randomly assigned using completely randomized design (CRD) to five treatments with seven replicates per treatment and seven birds per replicate. The birds were kept in battery cages with measurement of 120 cm (length) × 120 cm (width) × 45 cm (height). All the birds were subjected to brooding for 7 days at 31–32°C and gradually reduced the temperature by 2°C until it reached 25°C. The average relative humidity was between 60 and 75% throughout the study. For the house lighting, the birds were subjected to 24 h of light during the brooding and 8 h of light at night after the brooding period. The birds were fed with a starter diet from day 0 to day 21 and a finisher diet from day 22 to day 42 according to respective dietary treatments, namely, negative control (NC): basal diet only; OTC: basal diet + 0.01% oxytetracycline; RG11: basal diet + 0.1% Postbiotic RG11; RI11: basal diet + 0.1% postbiotics RI11 and RS5: basal diet + 0.1% postbiotic RS5 (Tables 1, 2). The basal diet was formulated using FeedLIVE software version 1.52 (Live Informatics Company Ltd., Thailand) according to the nutrient specification recommended by Cobb 500 Broiler Performance and Nutrition Supplement. All the birds were given feed, as shown in Tables 1, 2, and water *ad libitum*.

**Table 1:** Ingredient composition and nutrient contents of the starter diet.

**Ingredients (%)**	**Dietary treatments^a^**				
	**NC**	**OTC**	**RG11**	**RI11**	**RS5**
Corn	48.55	48.50	48.50	48.50	48.50
Soybean meal 48%	41.17	41.28	41.28	41.28	41.28
Palm oil	3.79	3.79	3.79	3.79	3.79
Wheat pollard	1.52	1.44	1.35	1.35	1.35
l-Lysine	0.06	0.07	0.07	0.07	0.07
DL-Methionine	0.31	0.32	0.32	0.32	0.32
MDCP 21%^b^	1.23	1.23	1.23	1.23	1.23
Calcium carbonate	1.78	1.78	1.78	1.78	1.78
Choline chloride	0.08	0.08	0.08	0.08	0.08
Salt	0.21	0.21	0.21	0.21	0.21
Mineral mix^c^	0.80	0.79	0.79	0.79	0.79
Vitamin mix^d^	0.30	0.30	0.30	0.30	0.30
Antioxidant^e^	0.10	0.10	0.10	0.10	0.10
Toxin binder^f^	0.10	0.10	0.10	0.10	0.10
Oxytetracycline^g^		0.01			
RG11			0.10		
RI11				0.10	
RS5					0.10
Total	100.00	100.00	100.00	100.00	100.00
**Calculated nutrient level^h^**					
ME kcal/kg^i^	3,010.98	3,009.65	3,009.64	3,009.64	3,009.64
Protein %	22.02	22.06	22.06	22.06	22.06
Fat %	5.87	5.87	5.87	5.87	5.87
Fiber %	4.21	4.23	4.20	4.20	4.20
Calcium %	0.98	0.98	0.98	0.98	0.98

a *NC: basal diet only (negative control), OTC: basal diet + 0.01% oxytetracycline (positive control), RG11: basal diet + 0.1% Postbiotic RG11, RI11: basal diet + 0.1% Postbiotic RI11, RS5: basal diet + Postbiotic RS5*.

b *Monodicalcium phosphate 21%*.

c *Mineral mix supplied per kg of feed: Co 0.6 mg, Cu 20 mg, Fe 100 mg, I 2 mg; Mn 110 mg, Se 0.2 mg, Zn 100 mg*.

d *Vitamin mix supplied per kg of feed: Vitamin A 11494IU, Vitamin D_3_ 1,725 IU, Vitamin E 40 IU, Vitamin K_3_ 2.29 mg, Cobalamin 0.05 mg, Thiamine 1.43 mg, Riboflavin 3.44 mg, Folic acid 0.56 mg, Biotin 0.05 mg, Pantothenic acid 6.46 mg, Niacin 40.17 mg, Pyridoxine 2.29 mg*.

e *Antioxidant comprises of butylated hydroxyanisole (BHA)*.

f *Toxin binder comprises natural hydrated sodium calcium aluminum silicates to bind to the mycotoxins present in the feed*.

g *Oxytetracyline (200 mg/kg, purity ≥ 64.7%, Y.S.P. Industries (M) SDN BHD*.

h *All the diets were formulated by using FeedLive International Software (Thailand)*.

i *ME kcal/kg = Metabolizable energy kcal/kg*.

**Table 2:** Ingredient composition and nutrient contents of the finisher diet.

**Ingredients (%)**	**Dietary treatments^a^**				
	**NC**	**OTC**	**RG11**	**RI11**	**RS5**
Corn	56.50	56.40	56.40	56.40	56.40
Soybean meal 48%	32.41	32.40	32.40	32.40	32.40
Palm oil	5.45	5.50	5.50	5.50	5.50
Wheat pollard	1.10	1.15	1.06	1.06	1.06
l-Lysine	0.02	0.02	0.02	0.02	0.02
DL-Methionine	0.24	0.24	0.24	0.24	0.24
MDCP 21%^b^	1.45	1.45	1.45	1.45	1.45
Calcium carbonate	1.20	1.20	1.20	1.20	1.20
Choline chloride	0.08	0.08	0.08	0.08	0.08
Salt	0.20	0.20	0.20	0.20	0.20
Mineral mix^c^	0.80	0.80	0.80	0.80	0.80
Vitamin mix^d^	0.30	0.30	0.30	0.30	0.30
Antioxidant^e^	0.10	0.10	0.10	0.10	0.10
Toxin binder^f^	0.15	0.15	0.15	0.15	0.15
Oxytetracycline^g^		0.01			
RG11			0.10		
RI11				0.10	
RS5					0.10
Total	100.00	100.00	100.00	100.00	100.00
**Calculated nutrient level^h^**
ME kcal/kg^i^	3,168.08	3,167.57	3,167.57	3,167.57	3,167.57
Protein %	18.78	18.74	18.74	18.74	18.74
Fat %	7.70	7.74	7.74	7.74	7.74
Fiber %	3.77	3.79	3.76	3.76	3.76
Calcium %	0.78	0.78	0.78	0.78	0.78

a *NC: basal diet only (negative control), OTC: basal diet + 0.01% oxytetracycline (positive control), RG11: basal diet + 0.1% Postbiotic RG11, RI11: basal diet + 0.1% Postbiotic RI11, RS5: basal diet + Postbiotic RS5*.

b *Monodicalcium phosphate 21%*.

c *Mineral mix supplied per kg of feed: Co 0.6 mg, Cu 20 mg, Fe 100 mg, I 2 mg; Mn 110 mg, Se 0.2 mg, Zn 100 mg*.

d *Vitamin mix supplied per kg of feed: Vitamin A 11494IU, Vitamin D_3_ 1725 IU, Vitamin E 40 IU, Vitamin K_3_ 2.29 mg, Cobalamin 0.05 mg, Thiamine 1.43 mg, Riboflavin 3.44 mg, Folic acid 0.56 mg, Biotin 0.05 mg, Pantothenic acid 6.46 mg, Niacin 40.17 mg, Pyridoxine 2.29 mg*.

e *Antioxidant comprises of butylated hydroxyanisole (BHA)*.

f *Toxin binder comprises natural hydrated sodium calcium aluminum silicates to bind to the mycotoxins present in the feed*.

g *Oxytetracyline (200 mg/kg, purity ≥ 64.7%, Y.S.P. Industries (M) SDN BHD*.

h *All the diets were formulated by using FeedLive International Software (Thailand)*.

i *ME kcal/kg = Metabolizable energy kcal/kg*.

### Growth Performance Measurement

The body weight (BW) and feed intake (FI) were taken weekly throughout the feeding trial. The collected data were used to calculate body weight gain (BWG) and feed conversion ratio (FCR).

### Samples Collection and Processing

A total of seven birds per treatment (one bird per cage) were randomly selected and slaughtered according to the Halal Slaughtering Protocol (21) at the age of 21 days (except for cecal microbiota and gene expression) and 42 days. The mucosa from the small intestine was flushed with cold phosphate buffer solution (0.01 M, pH 7.4), scraped using a glass slide, and stored in different 1.5 ml microcentrifuge tubes. The jejenum and cecum content were quickly collected and frozen in liquid nitrogen. All the samples were then stored at −80°C until analysis. Approximately 3–4 cm of each segment of the small intestine (duodenum, jejunum, and ileum) were collected and flushed with PBS (0.01 M, pH 7.4) before preserving in 10% (v/v) neutral buffered formalin at room temperature. The intestinal tissues were embedded in paraffin blocks and then cut into 4 μm using microtome before being placed on the glass slide.

### Mucosa Antioxidant

#### Glutathione Peroxidase

The glutathione peroxidase (GSH-Px) concentration in the intestinal mucosa was measured using chicken glutathione peroxidase (GSH-Px) ELISA kits (SunLong Biotech Co. LTD, China) by following the manufacturer's procedure.

#### Malondialchehyche

The malondialchehyche (MDA) concentration in the intestinal mucosa was measured using chicken malondialchehyche (MDA) ELISA kits (SunLong Biotech Co. LTD, China) by following the manufacturer's procedure.

#### Superoxidase Dismutase

The superoxidase dismutase (SOD) concentration in the intestinal mucosa was measured using chicken superoxidase dismutase (SOD) ELISA kits (SunLong Biotech Co. LTD, China) by following the manufacturer's procedure.

#### Crude Mucin Production

Crude mucin concentration in the excreta was measured 3 days consecutively before the sampling day proposed by Horn et al. (22) and Machado et al. (23). Fresh excreta were collected randomly from each replicate 2-times daily (8 am and 4 pm) in a Falcon tube and quickly chilled at 4°C. Before collection, a clean and the sanitized plastic canvas was placed under cages to collect fresh and excreta without contamination. Quantification assay was carried out using 3 g of excreta and added with chilled 25 ml of sodium chloride solution (0.15 M sodium chloride, 0.02 M sodium azide). The sample was immediately homogenized using Ultraturrax homogenizer for 1 min, centrifuged at 12,000 *g* for 20 min at 4°C, and the supernatant was decanted into a new, pre-weighed 50 ml Falcon tube. Later, 15 ml of chilled absolute ethanol was mixed with the supernatant. It was left overnight in the −20°C before centrifuging again at 12,000 *g* for 10 min at 4. The sediment or the pelleted mucin was rinsed again with 10 ml sodium chloride solution (0.15-M sodium chloride, 0.02-M sodium azide) and 15 ml of absolute ethanol, left overnight re-centrifuge until clear supernatant can be obtained. The supernatant was removed by aspiration and the sediment was collected and weighed as the crude mucin yield.

### Mucin Staining

#### Acidic Mucin

Acidic mucin produced by goblet cells was determined by using Alcian Blue (pH 2.5) according to Prophet et al. (24) with slight modification. Briefly, after deparaffinized and rehydrated with xylene and ethanol, the slides were stained in 8GX solution (pH 2.5) (Merck, 101647) for 30 min and washed under tap water for 10 min. Later, the slides were counterstained in a nuclear fast red solution (Sigma Chemical, 1.00121) for another 5 min, washed again in running tap water for 1 min before dehydration, cleared, and mounted with the coverslip. The goblet cell with acidic mucin stained blue while the nuclei stained red to pale pink.

#### Neutral Mucin

Neutral mucin was identified by periodic acid–Schiff (PAS) staining according to Fasina et al. (25). Sections were brought to water, placed in 0.5% periodic acid (Sigma Chemical, 1.00482) for 30 min, and washed in running tap water for 15 min. The slides were then immersed in Schiff's reagent (Sigma Chemical, S5133) for another 30 min before washing again for 10 min, dehydrated, and mounted with the coverslip. Neutral mucin-producing goblet cells stained pink as the result of the staining.

#### High Iron Diamine

The slides were stained in high iron diamine stain as described by Spicer (26) to distinguish between sulfomucin and sialomucin. The stain was prepared earlier by using 120 mg of *N*,*N*-Dimethyl-*m*-phenylenediamine dihydrochloride (Sigma Chemical, SA219223), and 20 mg of *N*,*N*-Dimethyl-*p*-phenylenediamine dihydrochloride (Sigma Chemical, D-5004) with 50 ml of distilled water. Then, mixed with 1.4 ml of 40% ferric chloride, the pH of the final solution should be around 1.5–1.6. The slides were deparaffinized and rehydrated using xylene and three different concentrations of alcohol. All the sections were stained in the prepared high iron diamine solution for 18 h, rinsed rapidly under running tap water for 30 min, immersed in Alcian Blue (Sigma Chemical, 66011) for 5 min, and dehydrated rapidly, clear, and mounted with a coverslip.

#### Morphometric Measurement

All the slides for each staining protocol were stained simultaneously in one batch to minimize any possible error. Every slide per histochemical staining for each intestinal segment, dietary treatment, and period of growth was viewed and evaluated under a light microscope (Leica RM2155, Germany) equipped with a digital camera (Leica DFC 295, Germany). A total of 10 villi and 10 crypts were examined and calculated for goblet cells per millimeter of villus height as described by Osho et al. (27).

### Intestinal Trefoil Factor

The intestinal trefoil factor (ITF) was determined using Chicken Intestinal Trefoil ELISA kits (QAYEE-BIO, China) according to the manufacturer's protocol. Briefly, the previously collected frozen intestinal tissues (1 g) were thawed on ice and homogenized with a 9 ml phosphate buffer solution (0.01 M, pH 7.4) to obtain the supernatant. A 10 μl of the collected supernatant was then added with 40 μl of diluent before being subjected to incubation 2-times. Finally, the concentration of ITF was calculated using the equation generated from the standard curve plotted.

### Deoxyribonucleic Acid Extraction and Ceca Bacteria Quantification

The DNA from the cecum content was extracted by using TriSure™ (Bioline, United Kingdom), homogenized using a vortex, and incubated for 3 min at room temperature. Later, 0.2 ml of chloroform was used to separate the sample into four phases (colorless upper phase, aqueous pale green, interphase, and organic sediment) by centrifuging at 12,000 *g* for 15 min at 4°C. All the layers were removed, leaving the interphase and organic layers before being added with 0.3 ml of absolute ethanol and centrifuged at 2,000 *g* for 5 min at 4°C. The precipitated DNA pellet was washed with 1 ml of 0.1-M sodium citrate in 10% ethanol and centrifuged again at 2,000 *g* for 5 min at 4°C. After two washes, the sample was added with 1.5 ml of 75% ethanol, incubated for 20 min at room temperature, and centrifuged at 2,000 *g* for 5 min at 4. Thereafter, the pellet was air dry for 15 min before being re-suspended with 8 mM of sodium hydroxide and centrifuged at 12,000 *g* for 15 min at 4°C. The supernatant was drawn out and transferred to a new sterile microcentrifuge tube and stored at −20°C for microbial quantification. The DNA concentration and purity were determined using BioSpectrometer^®^ basic (Eppendorf, Germany). Absolute quantification of bacteria in the sample was carried out by using real-time polymerase chain reaction (qPCR) and standard curves plotted with the known concentration of target bacterial DNA. Before that, a master mix (20 μl) was prepared by using 10 μl of 2x SensiFAST SYBR^®^ No-ROX Mix, 0.8 μl of each 10 μM forward and reverse primers, 2 μl sample, and 5.4 μl RNAase-free water. The reaction mixture was then analyzed using a CFX96 real-time PCR system (BioRad, Hercules, USA) with the cycling conditions as follows: polymerase activation at 95°C for 2 min, denaturation at 95°C for 5 s, annealing at 60°C for 10 s and followed by 72°C for 10 s. A melting curve was conducted to assess the product specificity in each amplification. The same PCR conditions were applied to all the target bacteria as shown in Table 3.

**Table 3:** The DNA primer sequences of target bacteria.

**Target bacteria**	**Primer sequence**	**Product size**	**References**
Total bacteria	F—CGGCAACGAGCGCAACCC R—CCATTGTAGCACGTGTGTAGCC	145	(28)
*Lactobacillus*	F—CATCCAGTGCAAACCTAAGAG R—GATCCGCTTGCCTTCGCA	341	(29)
*Bifidobacterium*	F—GGGTGGTAATGCCGGATG R—TAAGCCATGGACTTTCACACC	278	(30)
*Enterococcus* genus	F—CCCTTATTGTTAGTTGCCATCATT R—ACTCGTTGTACTTCCCATTGT	144	(28)
*Enterobacteriaceae*	F—CATTGACGTTACCCGCAGAAGAAGC R—CTCTACGAGACTCAAGCTTGC	195	(29)
*E. coli*	F—GTGTGATATCTACCCGCTTCGC R—AGAACGCTTTGTGGTTAATCAGGA	82	(29)

*F, Forward; R, Reverse*.

### Ribonucleic Acid Extraction and Gene Expression

For hepatic gene expression, the total RNA was extracted from 20 mg of liver tissue using innuPREP RNA Mini Kit 2.0 (Analytik Jena, Berlin, Germany) according to the manufacturer's protocol. The concentration and purity of the purified RNA were determined by using BioSpectrometer^®^ basic (Eppendorf, Germany) at the absorbance of 260/280 nm (ratio absorbance) prior converted to complementary DNA (cDNA) using SensiFAST™ cDNA Synthesis Kit. The relative mRNA levels of the genes were quantified through real-time PCR using the Bio–Rad CFX PCR system (Bio–Rad Laboratories, USA). The genes GADPH and β-actin were used as an endogenous control. Before running the qPCR, a master mix (20 μl) containing 10-μl 2X SensiFAST SYBR^®^ No-ROX Mix, 0.8-μl 10-μM forward primer, and 0.8 μl 10 μM reverse primer, 2-μl template and 6.4 μl RNAase-free water. Negative technical control without the presence of a template was used to verify the absence of contamination in the master mix. The list of gene primer sequences studied in this experiment is presented in Table 4. The qPCR cycling conditions were set using the following protocols: polymerase activation at 95°C for 2 min, denaturation of DNA at 95°C for 5 s, annealing at 60°C for 10 s, and extension at 72°C for 10 s. Analysis of the melting curve at the end of the amplification cycle was used to determine the product specificity. The relative gene expression was measured according to Livak and Schmittgen's (31) method.

**Table 4:** The sequence of target primers and housekeeping genes.

**Gene**	**Primer sequence (5'-3')**	**Product size (bp)**	**Accession number**
MUC 2	F-TTCATGATGCCTGCTCTTGTG R-CCTGAGCCTTGGTACATTCTTGT	93	NM_001318434.1
OCLN	F-ACGGCAGCACCTACCTCAA R-GGGCGAAGAAGCAGATGAG	123	XM_025144248
SIgA	F: GTCACCGTCACCTGGACTACA R: ACCGATGGTCTCCTTCACATC	192	S40610
GADPH	F-CTGGCAAAGTCCAAGTGGTG R-AGCACCACCCTTCAGATGAG	275	NM_204305

*F, Forward; R, Reverse; MUC 2, Mucin 2; OCLN, Occuludin; SIgA, Secretory Immunoglobulin A; GADPH, Glyceraldehyde-3-phosphate dehydrogenase*.

### Statistical Analysis

The collected data were analyzed using analysis of variance (ANOVA) in Statistical Analysis System (SAS), version 9.4, to determine differences between significant treatment means and followed by Duncan's Multiple Range Test where appropriate. The results were presented in means and standard error (SEM). The significant differences were declared at *p* < 0.05.

## Results

### Growth Performance

The growth performance of the broiler chickens is illustrated in Table 5. Although the results showed no significant difference in BW during the starter period, the birds fed with postbiotic RS5 showed significantly higher (*p* < 0.05) BWG than OTC fed birds. In the finisher period, OTC, RG11, and RS5 showed no significant difference (*p* > 0.05) in final BW and BWG. Although all treatment groups showed similarly (*p* > 0.05) FCR during starter, RS5 was revealed to have the lowest FCR compared to other dietary treatments. On the other hand, there was no significant difference (*p* > 0.05) recorded for feed intake in all dietary treatments throughout the study.

**Table 5:** Growth performance of chickens when fed with different postbiotics.

**Parameters**	**Treatment groups^1^**					**SEM^2^**	***p***
	**NC**	**OTC**	**RG11**	**RI11**	**RS5**		
**Starter (day 21)**							
Initial BW (g)	49.33	49.27	48.93	49.92	49.43	0.57	0.077
BW(g)	600.02^a^	595.85^a^	548.94^b^	566.35^ab^	601.56^a^	7.53	0.047
BWG (g)	552.70^a^	546.88^b^	499.01^c^	516.43^bc^	552.13^a^	14.50	0.022
FI (g)	952.76	976.22	903.67	903.60	949.26	5.23	0.56
FCR (g:g)	1.74	1.78	1.81	1.77	1.72	0.06	0.14
**Finisher (day 42)**							
Final BW(g)	2,351.20^ab^	2,259.00^ab^	2,258.65^ab^	2,205.43^b^	2,458.04^a^	32.72	0.042
BWG (g)	1,749.13^ab^	1,662.84^b^	1,709.71^ab^	1,639.08^ab^	1,856.46^a^	30.19	0.015
FI (g)	3,096.06	2,965.50	2,940.96	2,926.74	2,991.57	10.21	0.48
FCR (g:g)	1.77^a^	1.78^a^	1.72^a^	1.78^a^	1.63^b^	0.03	0.037

abc *Means with different superscripts in the same row differ significantly at p < 0.05*.

1 *Treatment groups: NC: basal diet only (negative control), OTC: basal diet + 0.01% oxytetracycline (positive control), RG11: basal diet + 0.1% Postbiotic RG11, RI11: basal diet + 0.1% Postbiotic RI11, RS5: basal diet + Postbiotic RS5*.

2 *SEM, Standard error of means*.

### Mucosa Antioxidant Concentration

#### Glutathione Peroxidase

The effects of the supplementation of *L. plantarum* postbiotics on GSH concentration in broiler chickens are shown in Table 6. When fed with postbiotics RI11, the chickens had significantly higher (*p* < 0.05) GSH concentration in the jejunum and ileum when compared to NC during the starter. However, no significant difference (*p* > 0.05) was observed in the duodenum. Meanwhile, in the duodenum during finisher, all the treatment groups showed no significant difference (*p* > 0.05) except for RS5. In the jejunum, the RI11 increased (*p* < 0.05) the enzyme concentration compared to other treatment groups. Also, the GSH concentration was lowest (*p* < 0.05) in NC, OTC, and RS5. There was no significant (*p* > 0.05) difference recorded in the ileum.

**Table 6:** The GSH-Px concentration of chickens when fed with postbiotics.

**Parameter**	**Treatment groups^1^**					**SEM^2^**	***p***
	**NC**	**OTC**	**RG11**	**RI11**	**RS5**		
**Starter (day 21)**							
Duodenum (ng/ml)	120.14	125.06	126.74	126.26	120.98	2.28	0.88
Jejunum (ng/ml)	120.14^c^	114.76^c^	120.86^c^	147.83^a^	133.94^b^	3.38	0.02
Ileum (ng/ml)	94.34^b^	123.02^a^	94.58^b^	119.42^a^	122.18^a^	4.10	0.004
**Finisher (day 42)**							
Duodenum (ng/ml)	65.37^ab^	78.14^a^	68.97^ab^	86.49^a^	55.67^b^	3.72	0.04
Jejunum (ng/ml)	74.94^c^	78.21^bc^	96.01^a^	92.40^ab^	71.85^c^	3.19	0.02
Ileum (ng/ml)	81.13	80.41	83.29	84.49	92.41	2.23	0.4

abc *Means with different superscripts in the same row differ significantly at p < 0.05*.

1 *Treatment groups: NC: basal diet only (negative control), OTC: basal diet + 0.01% oxytetracycline (positive control), RG11: basal diet + 0.1% Postbiotic RG11, RI11: basal diet + 0.1% Postbiotic RI11, RS5: basal diet + Postbiotic RS5*.

2 *SEM, Standard error of means*.

#### Malondialchehyche

Table 7 shows the MDA concentration of the broiler chickens fed with different postbiotics. The MDA concentration in both duodenal and ileal mucosa during the starter period had a significant difference (*p* < 0.05) among the treatment groups. In the duodenum, the highest concentration (*p* < 0.05) was recorded in NC and RG11, while NC, OTC, and RG11 recorded the highest concentration (*p* < 0.05) in the ileum. Unlike in the starter, there was no significant difference in MDA concentration in both duodenum and ileum. In the jejunum, dietary treatment RS5 had lowered (*p* < 0.05) the MDA concentration in the mucosal, whereas the NC had the highest MDA concentration.

**Table 7:** Malondialchehyche concentration of chickens when fed different postbiotics.

**Parameter**	**Treatment groups^1^**					**SEM^2^**	***p***
	**NC**	**OTC**	**RG11**	**RI11**	**RS5**		
**Starter (day 21)**							
Duodenum (mg/ml)	3.68^a^	3.06^b^	3.68^a^	2.80^b^	3.02^b^	0.178	0.028
Jejunum (mg/ml)	2.91	3.24	3.19	3.59	3.17	0.09	0.26
Ileum (mg/ml)	3.45^a^	3.50^a^	3.34^a^	3.05^b^	3.15^b^	0.05	0.0017
**Finisher (day 42)**							
Duodenum (mg/ml)	2.24	1.97	2.12	2.49	2.24	0.08	0.36
Jejunum (mg/ml)	2.54^a^	1.90^ab^	1.77^b^	1.50^b^	1.64^b^	0.012	0.01
Ileum (mg/ml)	1.94	1.75	1.79	1.63	1.72	0.18	0.12

abc *Means with different superscripts in the same row differ significantly at p < 0.05*.

1 *Treatment groups: NC: basal diet only (negative control), OTC: basal diet + 0.01% oxytetracycline (positive control), RG11: basal diet + 0.1% Postbiotic RG11, RI11: basal diet + 0.1% Postbiotic RI11, RS5: basal diet + Postbiotic RS5*.

2 *SEM, Standard error of means*.

#### Superoxidase Dismutase

The super-oxidase dismutase concentration of duodenum, jejunum, and ileum mucosa fed with different postbiotics is shown in Table 8. The OTC, RI11, and RS5 had higher (*p* < 0.05) SOD concentrations than other dietary groups in the jejunum during the starter. Meanwhile, the OTC and RS5 exerted the highest significant difference (*p* < 0.05) compared to the other groups in the ileum. During the finisher, OTC, RI11, and RS5 showed a significant difference in SOD concentration compared to NC and RG11 in the duodenum. The OTC had the lowest (*p* < 0.05) SOD concentration for jejunum compared to other treatment groups but showed no significant difference with NC and RI11. No significant difference (*p* > 0.05) was recorded in SOD concentration in the duodenum during starter and ileum during finisher.

**Table 8:** The SOD concentration of chickens when fed different postbiotics.

**Parameter**	**Treatment groups^1^**					**SEM^2^**	***P*** **-value**
	**NC**	**OTC**	**RG11**	**RI11**	**RS5**		
**Starter (day 21)**							
Duodenum (mg/ml)	17.06	15.58	17.44	18.11	17.54	0.39	0.35
Jejunum (mg/ml)	14.83^b^	17.69^a^	14.17^b^	17.52^a^	17.75^a^	0.47	0.02
Ileum (mg/ml)	15.11^b^	17.42^a^	13.78^b^	14.31^b^	18.53^a^	0.52	0.001
**Finisher (day 42)**							
Duodenum (mg/ml)	13.87^bc^	14.61^ab^	12.80^c^	16.33^ab^	17.11^a^	0.55	0.04
Jejunum (mg/ml)	13.20^b^	13.09^b^	16.51^a^	14.69^ab^	16.94^a^	0.51	0.01
Ileum (mg/ml)	12.5	14.13	14.18	13.35	14.72	0.32	0.22

abc *Means with different superscripts in the same row differ significantly at p < 0.05*.

1 *Treatment groups: NC: basal diet only (negative control), OTC: basal diet + 0.01% oxytetracycline (positive control), RG11: basal diet + 0.1% Postbiotic RG11, RI11: basal diet + 0.1% Postbiotic RI11, RS5: basal diet + Postbiotic RS5*.

2 *SEM, Standard error of means*.

### Crude Mucin Production

**Figure 1:**
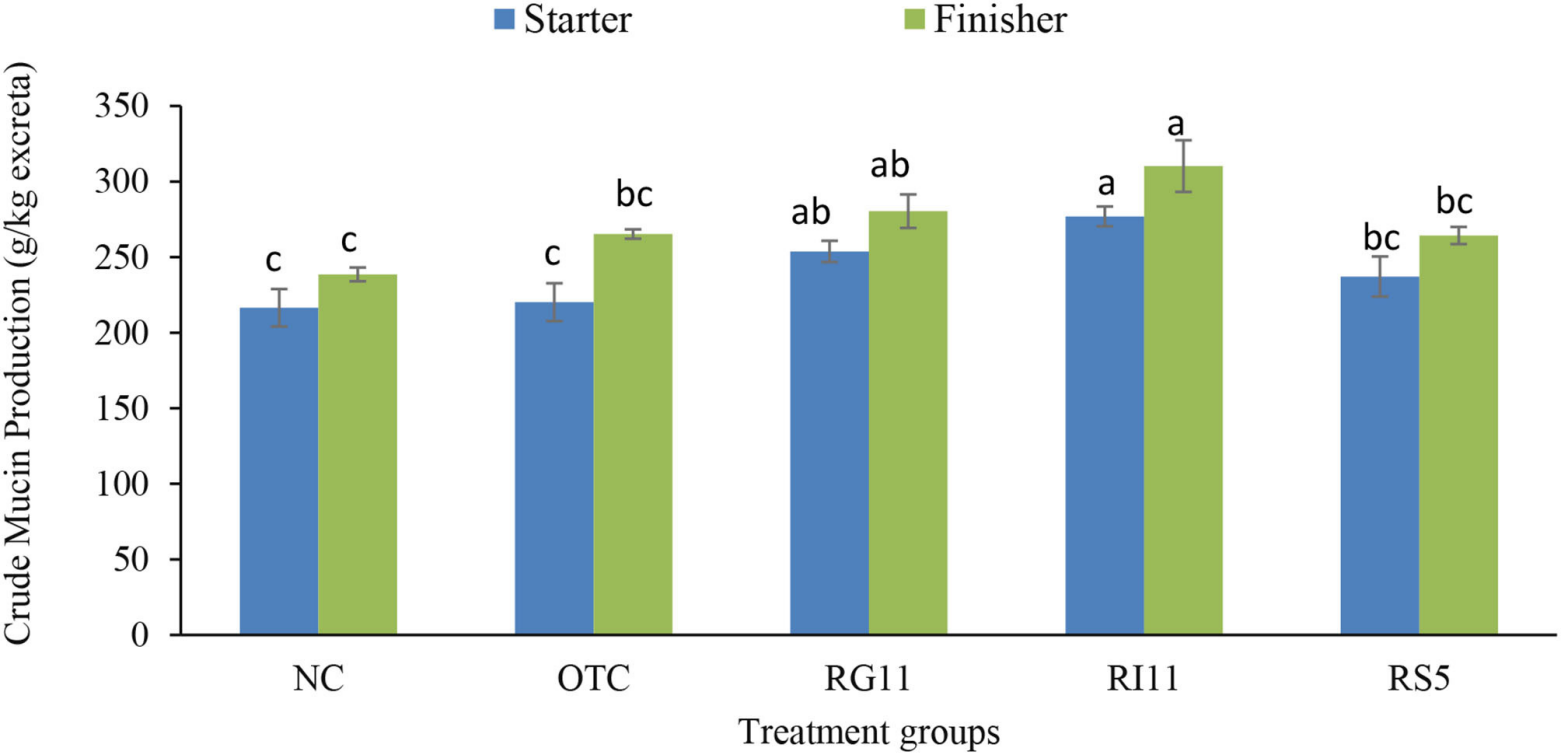
Quantification of crude mucin in broiler excreta when supplemented with different postbiotics. ^abc^Bar with different superscripts differ significantly at *p* < 0.05. Treatment groups: NC: basal diet only (negative control), OTC: basal diet + 0.01% oxytetracycline (positive control), RG11: basal diet + 0.1% Postbiotic RG11, RI11: basal diet + 0.1% Postbiotic RI11, RS5: basal diet + Postbiotic RS5.

The crude mucin production indicates mucin secretion, especially along the lower gastrointestinal tract. As illustrated in Figure 1, the crude mucin production was significantly highest in chickens fed with postbiotic RI11 even though it showed no significant difference with RG11. Meanwhile, the NC group recorded the lowest secretion of crude mucin.

### Mucin Staining

The effects of *L. plantarum* supplementation on mucin composition in the small intestine are illustrated in Table 9. The production of acidic mucin in the duodenum showed no significant difference (*p* > 0.05) in all the dietary treatment groups. However, the birds fed with postbiotic improved (*p* < 0.05) the production of acidic mucin in the jejunum and ileum compared to the OTC. There was no significant (*p* > 0.05) difference between all the treatments on the neutral mucin secretion in the goblet cells. No significant difference was observed in the sialomucin and sulfomucin production in the duodenum. The OTC group produced the lowest (*p* < 0.05) sulfomucin in the jejunum and ileum, even though it showed no significant difference (*P* > 0.05) with NC in the ileum. The supplementation of postbiotics impeded the production of sialomucin in both jejunum and ileum. However, no significant difference was found between RG11 and OTC in the jejunum. In the finisher period, as illustrated in Table 10, the inclusion of postbiotic in feed did not affect (*p* > 0.05) the acidic mucin secretion in the duodenum. On the other hand, the highest (*p* < 0.05) acidic mucin production was detected in RS5 in both jejunum and ileum, whereas NC had the lowest (*p* < 0.05) acidic mucin secretion. There was a significant difference (*p* < 0.05) between RS5 with NC and OTC groups in the duodenum and jejunum for the neutral mucin goblet cells. In the ileum, the supplementation of postbiotic and OTC did not influence (*p* > 0.05) the neutral mucin secretion in goblet cells. Moreover, in the duodenum, the RG11 had the highest (*p* < 0.05) sulfomucin production but no significant (*p* > 0.05) difference in other treatment groups. The RI11 and OTC produced the highest and lowest (*p* < 0.05) sulfomucin, respectively, in the jejunum. The secretion of sulfomucin in the ileum showed no difference (*p* > 0.05) between all the postbiotic treatment groups but was higher (*p* < 0.05) when compared with OTC. Moreover, no significant (*p* > 0.05) changes were detected in the sialomucin in the duodenum. Postbiotics RI11 and RS5 secreted the lowest (*p* < 0.05) sialomucin compared to NC and OTC groups in the jejunum. The RG11, RI11, and RS5 showed no significant difference (*p* > 0.05) in producing sialomucin in the ileum, but the lowest (*p* < 0.05) production was revealed in the NTC and OTC groups.

**Table 9:** Goblet cell mucin composition of broiler chickens when fed with different postbiotics during the starter period.

**Parameter**	**Treatment groups^1^**					**SEM^2^**	***p***
	**NC**	**OTC**	**RG11**	**RI11**	**RS5**		
**Acidic mucin (Numbers of gobbler cells/villus height in mm^2^)**							
Duodenum	1,304.75	1,281.25	1,321.5	1,342.5	1,317.5	22.67	0.46
Jejunum	1,594.25^ab^	1,531.75^b^	1,633.75^a^	1,634.25^a^	1,656^a^	15.23	0.043
Ileum	1,769^b^	1,744.75^b^	1,809^a^	1,870^a^	1,880.25^a^	28.51	0.012
**Neutral mucin (Numbers of gobbler cells villus height in mm^2^)**							
Duodenum	1,066.75	1,075.75	1,116.5	1,129.25	1,106.5	32.55	0.45
Jejunum	1,315	1,318.25	1,328	1,335	1,333	26.34	0.34
Ileum	1,439	1,450	1,469.75	1,465.75	1,494.25	26.98	0.28
**Sulfomucin (Numbers of gobbler cells/villus height in mm^2^)**							
Duodenum	1.33	1.30	1.34	1.35	1.37	0.39	0.66
Jejunum	1.411.46^b^	1.371.46^c^	1.43^ab^	1.46^a^	1.45^a^	0.03	0.022
Ileum	1.55^b^	1.54^b^	1.59^a^	1.58^a^	1.58^a^	0.012	0.103
**Sialomucin (Numbers of gobbler cells /villus height in mm^2^)**							
Duodenum	0.102	0.107	0.097	0.096	0.099	0.0019	0.49
Jejunum	0.166^a^	0.157^b^	0.157^b^	0.152^c^	0.15^c^	0.0027	0.002
Ileum	0.34^ab^	0.39^a^	0.29^b^	0.28 ^b^	0.26 ^b^	0.0087	0.016

abc *Means with different superscripts in the same row differ significantly at p < 0.05*.

1 *Treatment groups: NC: basal diet only (negative control), OTC: basal diet + 0.01% oxytetracycline (positive control), RG11: basal diet + 0.1% Postbiotic RG11, RI11: basal diet + 0.1% Postbiotic RI11, RS5: basal diet + Postbiotic RS5*.

2 *SEM, Standard error of means*.

**Table 10:** Goblet cell mucin composition of broiler chickens when fed with different postbiotics during the finisher period.

**Parameter**	**Treatment groups^1^**					**SEM^2^**	***p***
	**NC**	**OTC**	**RG11**	**RI11**	**RS5**		
**Acidic mucin (Numbers of gobbler cells/villus height in mm^2^)**							
Duodenum	1,002.33	1,039.25	1,084.33	1,148.17	1,107.67	19.25	0.11
Jejunum	1,166.58^b^	1,170.25^b^	1,254.33^b^	1,288.08^a^	1,262.33^ab^	17.70	0.047
Ileum	1,481.08^b^	1,485.5^b^	1,515.25^ab^	1,527.42^a^	1,521.67^ab^	20.2	0.024
**Neutral mucin (Numbers of gobbler cells /villus height in mm^2^)**							
Duodenum	1,000.33^b^	1,008.42^b^	1,003.58^b^	1,063.67^ab^	1,074.58^a^	30.92	0.046
Jejunum	1,162.75^b^	1,175.67^b^	1,217^a^	1,228.33^a^	1,218.75^a^	36.97	0.003
Ileum	1,265.17	1,243.42	1,268.83	1,268.5	1,289.58	22.61	0.56
**Sulfomucin (Numbers of gobbler cells/villus height in mm^2^)**							
Duodenum	0.65^ab^	0.66^ab^	0.74^a^	0.67^ab^	0.71^ab^	0.013	0.013
Jejunum	0.74^c^	0.73^c^	0.85^ab^	0.90^a^	0.81^bc^	0.01	0.02
Ileum	0.91^bc^	0.88^c^	1.02^a^	0.97^ab^	1.01^a^	0.015	0.0019
**Sialomucin (Numbers of gobbler cells/villus height in mm^2^)**							
Duodenum	0.085	0.096	0.091	0.099	0.094	0.006	0.964
Jejunum	0.106^a^	0.101^ab^	0.097^b^	0.099^b^	0.098^b^	0.001	0.033
Ileum	0.26^a^	0.26^a^	0.24^ab^	0.21^b^	0.20^b^	0.009	0.034

abc *Means with different superscripts in the same row differ significantly at p < 0.05*.

1 *Treatment groups: NC: basal diet only (negative control), OTC: basal diet + 0.01% oxytetracycline (positive control), RG11: basal diet + 0.1% Postbiotic RG11, RI11: basal diet + 0.1% Postbiotic RI11, RS5: basal diet + Postbiotic RS5*.

2 *SEM, Standard error of means*.

### Intestinal Trefoil Factor

The trefoil factor 3 or intestinal trefoil factor (ITF) in the duodenum, jejunum, and ileum of broiler chickens after being fed with *L. plantarum* postbiotics are shown in Table 11. The results showed a significant difference (*p* < 0.05) in ITF in different parts of the small intestine in both starter and finisher. During the starter diet period, no significant difference (*p* > 0.05) was observed among all the treatment groups in the duodenum. Moreover, the supplementation of RI11 and RS5 enhanced (*p* < 0.05) the ITF concentration in the jejunum. The ITF concentration was the lowest in the ileum (*p* < 0.05) for OTC and higher in postbiotic treatment groups. Interestingly, the NC had a significantly higher (*p* < 0.05) concentration than the OTC group in jejunum and ileum. Meanwhile, during the finisher, RS5 showed a significantly higher concentration (*p* < 0.05) than NC and RG11 but no significant difference (*p* > 0.05) between OTC and RI11 in the duodenum. On the other hand, NC and OTC exhibited a significantly lower (*p* < 0.05) concentration than RS5 in both jejunum and ileum.

**Table 11:** ITF activity of chickens when fed with different postbiotics.

**Parameter**	**Treatment groups^1^**					**SEM^2^**	***p***
	**NC**	**OTC**	**RG11**	**RI11**	**RS5**		
**Starter (day 21)**							
Duodenum (mg/ml)	1.11	1.15	1.06	1.21	1.01	0.02	0.40
Jejunum (mg/ml)	1.18^a^	0.99^b^	1.06^b^	1.23^a^	1.20^a^	0.03	0.002
Ileum (mg/ml)	1.18^b^	1.08^c^	1.33^a^	1.36^a^	1.30^a^	0.03	0.0002
**Finisher (day 42)**							
Duodenum (mg/ml)	0.63^bc^	0.72^ab^	0.55^c^	0.68^ab^	0.75^a^	0.02	0.01
Jejunum (mg/ml)	0.57^b^	0.57^b^	0.66^b^	0.82^a^	0.84^a^	0.03	<0.001
Ileum (mg/ml)	0.53^c^	0.63^bc^	0.64^abc^	0.69^ab^	0.81^a^	0.03	0.01

abc *Means with different superscripts in the same row differ significantly at p < 0.05*.

1 *Treatment groups: NC: basal diet only (negative control), OTC: basal diet + 0.01% oxytetracycline (positive control), RG11: basal diet + 0.1% Postbiotic RG11, RI11: basal diet + 0.1% Postbiotic RI11, RS5: basal diet + Postbiotic RS5*.

2 *SEM, Standard error of means*.

### Ceca Bacterial Quantification

The microbial population supplemented with selected L. plantarum postbiotics in the cecum is shown in Table 12. For the total bacteria, the supplementation of L. plantarum postbiotics had improved (p <0.05) all the bacteria population except Lactobacillus. The results indicated that RS5 had a higher (p <0.05) count than NC. Meanwhile, the Bifidobacterium was found significantly higher (p <0.05) in the postbiotic treated groups compared to the NC but no significant difference (p > 0.05) with OTC. The inclusion of OTC and postbiotics RG11, RI11, and RS5 in the feed reduced (p <0.05) the colonization of ENT and E. coli in the caecum of broiler chickens. For Enterococcus, RI11 and NC recorded the highest and lowest (p <0.05) populations in the caecum, respectively.

**Table 12:** Ceca microbial quantification of chickens when fed with different postbiotic.

**Parameter** **(log_**10**_ CFU/g)**	**Treatment groups^1^**					**SEM^2^**	***p***
	**NC**	**OTC**	**RG11**	**RI11**	**RS5**		
Total bacteria	9.05^b^	9.37^ab^	9.23^ab^	9.14^ab^	9.52^a^	0.06	0.014
*Lactobacillus*	6.73	6.59	6.66	6.79	7.03	0.09	0.68
*Bifidobacterium*	7.01^c^	7.61^ab^	7.88^a^	7.84^a^	7.92^a^	0.11	0.038
Enterobacteriaceae	6.39^a^	5.16^b^	5.33^b^	5.44^b^	5.17^b^	0.12	0.0002
*E. coli*	6.49^a^	6.02^b^	5.79^b^	5.78^b^	5.80^b^	0.07	0.0002
*Enterococcus*	5.36^c^	7.36^ab^	7.28^b^	7.82^a^	7.68^ab^	0.22	<0.0001

abc *Means with different superscripts in the same row differ significantly at p < 0.05*.

1 *Treatment groups: NC: basal diet only (negative control), OTC: basal diet + 0.01% oxytetracycline (positive control), RG11: basal diet + 0.1% Postbiotic RG11, RI11: basal diet + 0.1% Postbiotic RI11, RS5: basal diet + Postbiotic RS5*.

2 *SEM, Standard error of means*.

### Gene Expression

**Figure 2:**
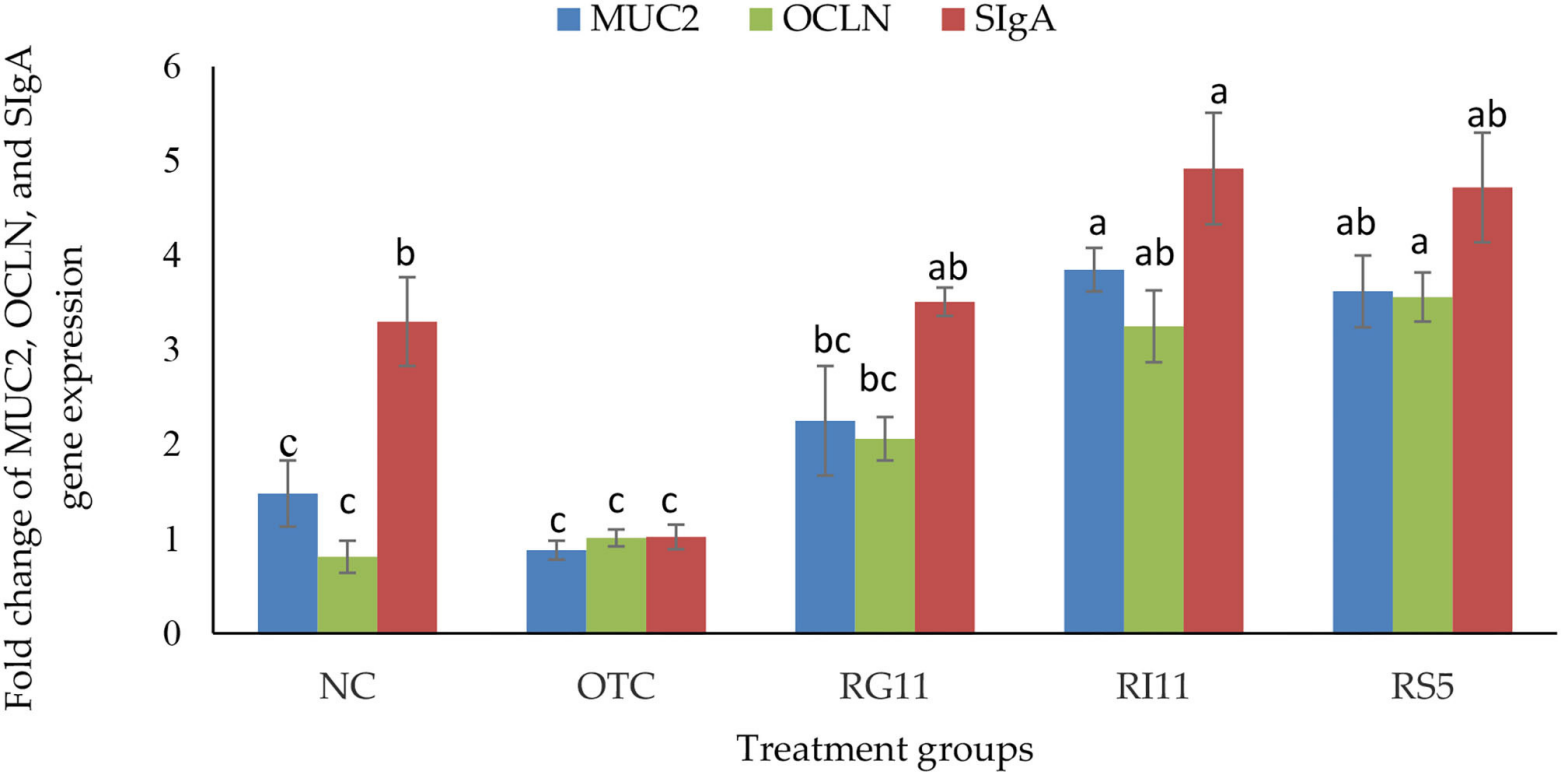
Gene expression of jejunal MUC2, OCLN, and SIgA in broiler chickens when supplemented with different postbiotics. ^abc^Bar with different superscripts differ significantly at *p* < 0.05. Treatment groups: NC: basal diet only (negative control), OTC: basal diet + 0.01% oxytetracycline (positive control), RG11: basal diet + 0.1% Postbiotic RG11, RI11: basal diet + 0.1% Postbiotic RI11, RS5: basal diet + Postbiotic RS5.

The result for the expression of SIgA, MUC2, and OCLN genes is shown in Figure 2. The gene expression of SIgA in broiler chickens was the highest (*p* < 0.05) in RI11, the lowest (*p* < 0.05) in OTC, but no significant difference (*p* > 0.05) was found between all the postbiotic treated groups. The supplementation of postbiotic RI11 had up-regulated (*p* < 0.05) the expression of MUC2 than other treatment groups despite showing no significant difference (*p* > 0.05) with RS5. Moreover, the expression of the OCLN gene was up-regulated (*p* < 0.05) by the supplementation of RS5 and RI11 compared to the NC and OTC groups.

## Discussion

### Growth Performance

Postbiotics are the bioactive secondary metabolites produced from lactic acid bacteria during growth and metabolism, which has similar positive effects to probiotics but without the inclusion of bacterial cells (32, 33). The postbiotic produced from L. plantarum comprised many beneficial compounds such as antioxidant compounds, organic acids, bacteriocins, and enzymes (16, 20). As a result, the postbiotics modulate the immune response, fight against pathogenic infections, stimulate the proliferation of good bacteria in the gut, and improve livestock growth performance and production (7, 14, 17). The production of plantaricin Ef and plantaricin W bacteriocin, as well as SCFAs such as acetic acid, caproic acid, and lactic acid, are well known for their broad spectrum of antimicrobial and anti-inflammatory activities, involved in intestinal health, particularly tight junction and mucous production (34–37). Chang et al. (16) revealed that the *L. plantarum* postbiotics also produced acetoin, intermediary compounds, and pyrrole compounds post bacterial fermentation. These compounds exert inhibitory activity against various pathogens and prevent the occurrence of inflammation and oxidation in the host.

Postbiotics enhance the growth performance through several postulated mechanisms when supplemented to the birds. The SCFA and bacteriocin in the postbiotics exerted bacteriostatic and bactericidal properties against pathogens in the gastrointestinal tract. Moreover, the acidic property of SCFA lowered the gut pH, which has a negative correlation with the proliferation of low acidic tolerant pathogenic bacteria such as Enterobacteriaceae, E. coli, and Salmonella (38). Such events also stimulated the proliferation of beneficial microflora in the gut, such as Lactobacillus and Bifidobacteria, to produce various positive implications on the immune system, digestive health, and growth performance in the birds. Furthermore, the reduced pathogen load also reduced the chances in adherence to the intestinal epithelial cell and preventing infection. It is also presumed that the SCFAs produced by commensal bacteria, particularly propionate and butyrate, have numerous health-promoting effects, including decreasing the risk of gut leakage, improving gut immunity, stimulating anti-inflammatory cytokines, and modulating the cellular activity of gut epithelial cells (39, 40). Most studies revealed that the supplementation of postbiotics improved villus height and crypt depth (17, 41–43). Both parameters are the general indication of good intestinal health, epithelial cell turnover, and nutrient absorption across the intestine into the systemic circulation (44, 45). All the evidence suggests that the *L. plantarum* postbiotic mechanism of action promoted positive growth performance in poultry.

### Mucosal Antioxidant Concentration

Recently, there has been a growing interest in the oxidative stress and antioxidant capacity of the supplement used in the animal feed due to heat stress and low-grade feedstuffs (feed toxin) (46, 47). As a result, these stressors are predisposing factors for disruption of normal physiological responses, which involve molecular and cellular activities or better known as inflammation (46, 48). The interaction between the microbes such as *Eimeria* and their metabolite with mucosa can lead to oxidative stress by forming reactive oxygen species (ROS) and destroying the intestinal epithelial barrier and the tight junction. In turn, this resulted in poor growth performance and nutrient absorption (49, 50). Although stressors are inevitable, it is crucial to include natural antioxidants feedstuffs to promote the animals' antioxidant capability, particularly in the gut. For example, several studies suggested that *L. plantarum* postbiotic (cell-free supernatant) has antioxidant activity, significantly inhibits pathogen growth, and downregulated the expression of IL-8 in human HT-29 cells (16, 51, 52).

In the intestinal mucosa, the enzymatic defense system such as GSH-Px and SOD protects the host against oxidative stress (53) apart from the antioxidant substance system; for example, lipid-soluble and water-soluble antioxidants (47). This study showed that the postbiotics produced by *L. plantarum* enhanced the antioxidant concentration of GSH-Px and SOD in the birds while lowering MDA in the intestinal mucosa. Research conducted by Humam et al. (17) and Izuddin et al. (42) highlighted that the *L. plantarum* postbiotics significantly enhanced the total antioxidant capacity, catalase, glutathione, GSH-Px, and thiobarbituric acid reactive substance activities in broiler chickens and post-weaning lambs. Both GSH-Px and SOD are highest at the early growth and intestinal development (47). In other words, the supplementation of postbiotics played a protective role during the crucial stage of growth to fully develop the intestinal tract against oxidative stress. In addition, MDA is the lipid peroxidation marker that indicates oxidative stress and is associated with cell membrane damage. In this study, however, the birds fed with postbiotics RI11 and RS5 significantly reduced the MDA level in the small intestine compared to the control groups. This agrees with Zhang et al. (54) and Jiang et al. (55) that high levels of GSH-Px and SOD would reduce MDA levels because both enzymes can remove free radicals generated by MDA.

### Intestinal Health, Tight Junction Permeability, and Mucin Production

Good intestinal health is essential for the growth and production of poultry. The intestinal tract has the largest mucosal tissue and secreted numerous immune-associated cells, including mast cells, goblet cells, secretory IgA (SIgA), and other epithelial-derived factors. All these cells contribute to various physiological and immunological reactions in the body to prevent the adhesion of pathogens on the intestinal epithelial cell and maintain health (56). Under normal conditions, the intestinal epithelium, together with mucin secretion and tight junction proteins, selectively permit the passage of nutrients, ions, and water but hinder the entrance of infectious agents and feed toxins by forming protective and functional barriers (57, 58). Furthermore, the mucus secreted by goblet cells prevents mechanical injury, bacterial translocation, and maintains intestinal equilibrium. As the major component of mucus besides carbohydrates, lipids, water, and proteins, the mucin, particularly acidic mucin, and sulfomucin, enhance intestinal defense (59, 60). Moreover, mucin is also the source of carbohydrates for the commensal bacteria (61). In other words, once the intestinal equilibrium has collapsed, the birds are prone to be infected with pathogenic infections.

From the findings, crude mucin production is the general indicator of mucus production along the gastrointestinal tract. To the best of our knowledge, this is the first study to evaluate the effect of the supplementation of postbiotics on crude mucin production. However, the significantly higher crude mucin production in birds treated with postbiotic RI11 might be associated with goblet cell density and MUC2 secretion in the intestine (62). The findings revealed that the birds fed with *L. plantarum* postbiotics RI11 and RS5 produced significantly higher acidic mucin and sulfomucin in the small intestine. The higher secretion of acidic and sulfated mucin protects the host epithelial layers against bacterial invasion. Both types of mucin are more resistant to bacterial glycosidase and host protease (63). Hence, reduction of the sulfomucin could affect the mucus layer and intestinal health. The production of the sialomucin has been implicated with changes in the intestinal microbiota (64). In the mice model, the infection caused by rotavirus changed the mucin sulfation and sialylation, while *Pseudomonas aeruginosa*-infected mice were found to have higher sialic mucin in the bronchial mucosa (65). However, more experiments have to be performed to fully understand the possible relationship between the sulfomucin and sialomucin in the intestine, especially during inflammation in poultry. Remarkably, the secretion of the neutral mucin is more prominent in the gastric mucosa, while the acidic mucin is abundantly found in the intestinal mucosa (66). This supported the finding of this study where a lower number of goblet cells with the neutral mucin than acidic mucin in the small intestine.

The supplementation of postbiotics promoted intestinal trefoil factor secretion in the small intestine, which is important for mucosal healing and against oxidation injury on epithelial cells (67). Although no clear explanation has been reported on the effect of ITF in poultry, in mice, ITF disrupted genes hindered the repairing of the mucosal lining and led to mortality due to colitis (68). Another study conducted by Wang et al. (69) revealed that burn-induced injury mice treated with recombinant human ITF (rhITF) protected the intestinal mucosal lining, and promoted the production of acidic mucin and sulfated mucin while lowering the secretion of neutral mucin. Moreover, the control group mice also had lower villi height, necrosis, and lymphocyte infiltration compared to rhITF mice. A similar finding was reported by Hu et al. (70), where ITF significantly reduced the damage to the endoplasmic reticulum and glutamine transporters on the intestinal epithelial cells. To our best knowledge, the finding from this study showed that the supplementation of postbiotics promoted ITF production in the intestinal mucosal, particularly in broiler chickens. Consequently, it can be postulated that ITF exhibits cryoprotective activity via continuous secretion of acidic mucin by goblet cells even though inflammation and oxidative stress are inevitable in poultry farming nowadays.

Concurrently, the presented data showed that L. plantarum postbiotic improved intestinal permeability by up-regulating the expression of OCLN in the jejunum. The previous study had demonstrated that the supplementation of yeast and probiotic L. plantarum 16 (Lac 16) up-regulated the intestinal barrier-related genes such as OCLN and MUC2 (71). Studies on mice documented that OCLN knockout mice showed chronic inflammation and hyperplasia in the gastric epithelium and testicular atrophy, albeit had normal barrier function (72, 73). The reduction of OCLN gene expression is also associated with various intestinal inflammatory diseases. Thus, this suggests that it has a vital role in maintaining intestinal barrier integrity (74).

Lactic acid bacteria are well recognized as immunostimulants associated with cytokine production, which subsequently affect the innate and adaptive immune responses (75). Secretory IgA protects the mucosal surface in the mucosa by presenting the bacterial antigens to dendritic cells. Together with mucous, trefoil peptides, and resistin-like molecule β, these compounds form a polarized and tight barrier on the intestinal epithelium (74). Besides that, SIgA also protects the intestinal epithelial cell from bacterial toxins and regulates mucosal homeostasis (76). In this study, the up-regulation of SIgA in postbiotic treated groups. Other studies have liked that the supplementation of L. plantarum increased the level of SIgA despite was the animals were being challenged by Clostridium perfringens and Salmonella pullorum (77). The up-regulation of SIgA also helped to modulate the gut microbiota as it binds to the commensal bacteria in the gut (78). Therefore, this explained that the postbiotic treated birds had a higher population of good bacteria in the caecum, subsequently improving the birds' nutrient performance.

### Ceca Microbiota

The gastrointestinal microbiota is inextricably linked to general health, which is not only responsible for digestion and absorption but also indirectly involves the endocrine and immune systems. Any changes in the feed composition, nutrients, and medication, particularly antibiotics, could influence the gut microbiota composition. Meanwhile, caecum harbors the most complex microbial community to prevent pathogen load, digest non-starch polysaccharides, involves in the detoxification process, and produce and absorption of nutrients such as amino acids (79, 80). There is no strong evidence of a healthy microflora pattern in the gut so far till this study took place. However, the research findings proved that the modulation of gut microbiota is highly associated with the production and performance of the host through the secretion of its metabolites and synergetic reaction with the internal environment (81, 82).

In this study, the supplementation of L. plantarum had up-regulated the population of beneficial bacteria such as Bifidobacterium and Enterococcus. These normal inhabitants in the gut are proven to stimulate the proliferation of commensal bacteria through secreting bacteriocin and organic acids to kill (or inhibit) the bad bacteria from the external environment (83, 84). Moreover, the inclusion of organic acids and bacteriocin from the postbiotic lowered the microenvironment pH and prohibited the colonization of ENT and E. coli due to cell lysis (85, 86). This is further proven by Chang et al. (16). The high concentration of acetic acid impeded the growth of Salmonella enterica CS3 and Listeria monocytogenes L55, and caproic acid exerted bacteriostatic activity against E. coli E-30, L. monocytogenes L55, and vancomycin-resistant enterococcus (VRE). A study conducted by Humam et al. (17) also found that the low cecal pH negatively correlates with the pathogenic bacteria count. In other words, the lower the cecal pH, the lower the population of ENT, E. coli, and Salmonella in the caecum. A similar result can be seen in Kareem et al. (87), the supplementation of postbiotic with inulin significantly reduced the population of E. coli and ENT compared to the control groups. In return, the commensal bacteria provide several primary benefits to the host through the competitive exclusion of pathogens or non-indigenous microbes, immune stimulation and modulation, and influence the nutrient digestion and absorption of the host (88). Under this condition, even if pathogens breach the immune system, the risk of getting an infection is minimized through immunomodulation in the gut, particularly the intestine.

Furthermore, a healthy gut microbiota composition regulates the mucin secretion, tight barrier junction, and epithelial cell turnover; hence, reducing the pathogens' chance of adhesion in the intestinal cells. In terms of immunity, the production of IgA, the predominant immunoglobulin in the intestine, inhibits the bacterial and viral adhesion to the epithelial cells and neutralizes toxins (89). Similar to other physiological reactions in the body, these processes require feed rich in energy and protein; therefore, balance and nutritious feed influence the gut microbiome composition. Poor quality (and quantity) of the feed may increase inflammatory responses, dysbiosis, and often reduce the growth performance of the livestock. However, the study on the cecal and intestine microbiome composition via genomic sequencing should be carried out in the future to explore the impact of feeding postbiotics on the variation of gut microbiota.

## Conclusion

In conclusion, postbiotic RS5 improved growth performance, antioxidant concentration, and acidic and MUC2 production by reducing intestinal integrity and bacteria. Hence, *L. plantarum* postbiotic is a good candidate to substitute AGP in poultry feed with promising beneficial effects on growth performance, antioxidant concentration, mucin production, gut permeability, microbiota, and immune response.

## Data Availability Statement

The raw data supporting the conclusions of this article will be made available by the authors, without undue reservation.

## Ethics Statement

The animal study was reviewed and approved by Institutional Animal Care and Use Committee (IACUC) of the Universiti Putra Malaysia (Protocol No: UPM/IACUC/AUP-R085/2018).

## Author Contributions

HC, TL, and HF were involved in the experimental design. HC, TL, and EL carried out the feeding trial, sample collection, and data analysis. All authors finally read and approved this manuscript to be published.

## Funding

This work was funded by Fundamental Research Grant Scheme (FRGS/1/2017/WAB01/UPM/01/1) received from the Malaysia Ministry of Higher Education.

## Conflict of Interest

The authors declare that the research was conducted in the absence of any commercial or financial relationships that could be construed as a potential conflict of interest.

## Publisher's Note

All claims expressed in this article are solely those of the authors and do not necessarily represent those of their affiliated organizations, or those of the publisher, the editors and the reviewers. Any product that may be evaluated in this article, or claim that may be made by its manufacturer, is not guaranteed or endorsed by the publisher.
